# Detecting cerebrovascular changes in the brain caused by hypertension in atrial fibrillation group using acoustocerebrography

**DOI:** 10.1371/journal.pone.0199999

**Published:** 2018-07-06

**Authors:** Wioletta Dobkowska-Chudon, Miroslaw Wrobel, Pawel Karlowicz, Andrzej Dabrowski, Andrzej Krupienicz, Tomasz Targowski, Andrzej Nowicki, Robert Olszewski

**Affiliations:** 1 District Hospital, Cardiology, Radom, Poland; 2 Sonovum AG, Leipzig, Germany; 3 Sonomed Sp. Z o.o., Warsaw, Poland; 4 MTZ Clinical Research, Warsaw, Poland; 5 Medical University of Warsaw, Warsaw, Poland; 6 Department of Geriatrics, National Institute of Geriatrics, Rheumatology and Rehabilitation, Warsaw, Poland; 7 IPPT, Polish Academy of Science, Department of Ultrasound, Warsaw, Poland; Indiana University, UNITED STATES

## Abstract

Acoustocerebrography is a novel, non-invasive, transcranial ultrasonic diagnostic method based on the transmission of multispectral ultrasound signals propagating through the brain tissue. Dedicated signal processing enables the estimation of absorption coefficient, frequency-dependent attenuation, speed of sound and tissue elasticity. Hypertension and atrial fibrillation are well known factors correlated with white matter lesions, intracerebral hemorrhage and cryptogenic stroke numbers. The aim of this study was to compare the acoustocerebrography signal in the brains of asymptomatic atrial fibrillation patients with and without hypertension. The study included 97 asymptomatic patients (40 female and 57 male, age 66.26 ± 6.54 years) who were clinically monitored for atrial fibrillation. The patients were divided into two groups: group I (patients with hypertension) n = 75, and group II (patients without hypertension) n = 22. Phase and amplitude of all spectral components for the received signals from the brain path were extracted and compared to the phase and amplitude of the transmitted pulse. Next, the time of flight and the attenuation of each frequency component were calculated. Additionally, a fast Fourier transformation was performed and its features were extracted. After introducing a machine learning technique, the ROC plot of differentiations between group I and group II with an AUC of 0.958 (sensitivity 0.99 and specificity 0.968) was obtained. It can be assumed that the significant difference in the acoustocerebrography signals in patients with hypertension is due to changes in the brain tissue, and it allows for the differentiating of high-risk patients with asymptomatic atrial fibrillation and hypertension.

## Introduction

Hypertension (HT) is a disease widespread in society, affecting 85.7 million adults in the United States and 1 billion individuals worldwide. It is the predominant cause of global disease prevalence and overall health loss [1, 2].

Lesions of the brain are very common in hypertensive patients. Age and systolic blood pressure are independent predictors for asymptomatic cerebrovascular damage, even in the absence of neurologic abnormalities [3, 4].

Silent brain infarcts and white matter lesions (WML) are detected by neuroimaging in approximately 10–20% of elderly patients in population-based studies [5, 6, 7]. Moreover, hypertension is a potentially modifiable risk factor and is the predominant cause of deep cerebral-micro-bleeds (CMB) [8]. Furthermore, HT is the most important known risk factor associated with the development of atrial fibrillation (AF) [9, 10]. Change in the shape and wall structure of the atrium due to dysfunction in the renin-angiotensin-aldosterone system is one of the explained mechanisms underlying AF [11]. Hypertension may lead to atrial fibrillation, as well as increased mortality, in patients with AF [12, 13]. Atrial fibrillation alone is a common, and treatable, risk factor for stroke [14]. AF and hypertension together induces not only ischemic strokes, but also CMB. Hypertension and AF are well known factors correlated with WML numbers. In the general population, around 10–20% of atrial fibrillation patients may also have CMB, and this increases to 30% in AF patients with a prior stroke [15]. One systematic review proved that while overall stroke risk seems to be double in patients with CMBs, the risk for intra-cerebral-hemorrhage (ICH) increased up to eight-fold [16]. More recently, diffuse WMD and micro-brain destruction have come to be recognized as an important cause of cognitive decline and dementia [17]. Advances in the access to, and in the performance of, brain magnetic-resonance-imaging (MRI) and computed tomography (CT) has led to an increased detection of asymptomatic abnormalities in the brains of patients with cardiovascular diseases. Both these methods are costly and have some well-known limitations [18]. These limitations already show the need for some alternative solution for AF and HT patients. ACG is a novel technique that captures the states of human brain tissue and its changes on the cellular level. It is based on noninvasive measurements of various parameters obtained by analyzing an ultrasound pulse emitted across the human skull [19]. In our previous study, we showed that ACG is an effective method for brain examination and detecting WML in the brains of patients with asymptomatic atrial fibrillation as compared to MRI [20]. We also proved that ACG allows for early detection of changes in the brains of patients with HT, when compared to healthy patients, which we presented at the European Society of Cardiology Congress in Barcelona in 2017.

The main task of our study was to differentiate the acoustical parameters measured in the brain, using ACG techniques in patients with asymptomatic atrial fibrillation, both with and without hypertension.

## Materials and methods

Ninety-seven patients were recruited in the study. Our patients were ambulatory, enrolled from among subjects who were surveyed in the clinical research for atrial fibrillation. The study included 40 female and 57 male patients (age 66.26 ± 6.54 years), with documented persistent or paroxysmal AF. Patients with congestive heart failure, previous myocardial infarction, or a history of symptomatic cerebrovascular accident were excluded from the study. Data included demographic and clinical characteristics, medical history, smoking habits, alcohol abuse, dyslipidemia, heart rhythm history, and pharmacologic treatment. The patients were divided retrospectively into two groups: group I (patients with HT) n = 75, and group II (patients without HT) n = 22. Hypertension was defined as systolic BP ≥140 mmHg, diastolic BP ≥90 mmHg, or the use of antihypertensive medication.

This study was performed in accordance with the Declaration of Helsinki and approved by the Human Investigation Review Committee at the Warsaw Medical Chamber. Each participant provided written informed consent to participate in this study.

We have introduced to our study a non-invasive ACG method based on acoustic spectroscopy, which examines the cellular and molecular brain structure. The technique allows the states and changes of human brain tissue to be captured [19]. The ACG uses multifrequency ultrasound pulses to detect and classify possible undesirable cellular, or molecular, changes in the brain tissue. The evaluation of the measured data is adjusted by means of statistical methods.

Basically, the signal transmitted through the skull is a compound pulse comprising of *n* discrete frequencies from f_1_ to f_n,_ sampled with frequency F_s_ >> f_n_. The spectrum of the received signal is modified by specific acoustical properties of the examined brain tissue, especially by the dispersion of the different frequencies. The individual amplitudes of spectral components deliver the information on absorption/attenuation of ultrasound along the propagation path and are extracted from the acquired pulses using the Least Square Method. Simultaneously, the phase angles of all received frequency components are calculated in order to estimate the time of flight of the pulse, for each of *n* frequency components, required to reach the receiver.

In short, ACG is based on noninvasive measurements of various parameters obtained by analyzing ultrasound pulses propagating along the human brain. The main idea of this method relies on the relation between the tissue density *p*, bulk modulus *K* and speed of sound *c*, of the tissue under examination. The most important parameters estimated in the ACG method are: attenuation, absorption coefficient, frequency-dependent attenuation, speed of sound and tissue elasticity. Speed of sound or, equivalently, times of arriving (ToA) of pulses propagating along the brain path, can be inferred from phase relations between spectral components of the received spectra [19, 20]. Basically, the ToA for the transmitted pulse through a skull is calculated from transmission/reception phases for two sine bursts with carrier frequencies f_1_ and f_2_, respectively. This rather elementary idea was modified by introducing a new multifrequency (10 components within the 1.3 MHz bandwidth, from 0.7 to 2 MHz) transmitting/receiving system that considerably improved the precision of the estimations of velocities and attenuations in intra-cranial tissue. The phase and amplitude, for all 10 frequency components, of the received signals from the brain path were extracted and compared to the phase and amplitude of the transmitted pulse, with the precision of 0.1° to 1° for phase and 5.6 ns for estimation of time of flight. This results in very high precision measurements of speed of sound in brain tissue, *d*c = 1.25 m/s, and changes in local tissue density, *dp*/*p* = 1.6·10^−3^. The bulk elasticity modulus *Κ* is calculated with a precision exceeding *d Κ / Κ* = 2.52·10^−5^.

First, we are extracting phase and amplitude from raw ultrasonic signals. In the next step, the data are split into 10-second intervals and the calculations of the time of flight and attenuation are performed. After normalization of all variables, the Artificial Neural Networks was constructed. Artificial Neural Networks are already shown to be effective in image [21] and voice [22] recognition. Both a vanilla deep neural network (using only fully connected layers) and a deep convolutional network (using fully connected and convolutional layers) were implemented. ReLU6 and cReLU were used as activation functions. The network training was conducted using an ADAM optimizer and backpropagation and was built upon the TensorFlow [23] framework in Python 3 software.

Evaluation of the network was done with a 4-fold cross-validation.

All three records for the patient were used—without rejection, the median was taken into account—the medical experiment was carried out reliably, and signals of a fixed quality were recorded. This qualitative criterion (over 70%) displayed by the device corresponds to the integrated energy of the received signal for the two extreme frequencies, after the initial selection of the transmit amplitude for individual components (in the range from 1 to 255).

**Fig 1:**
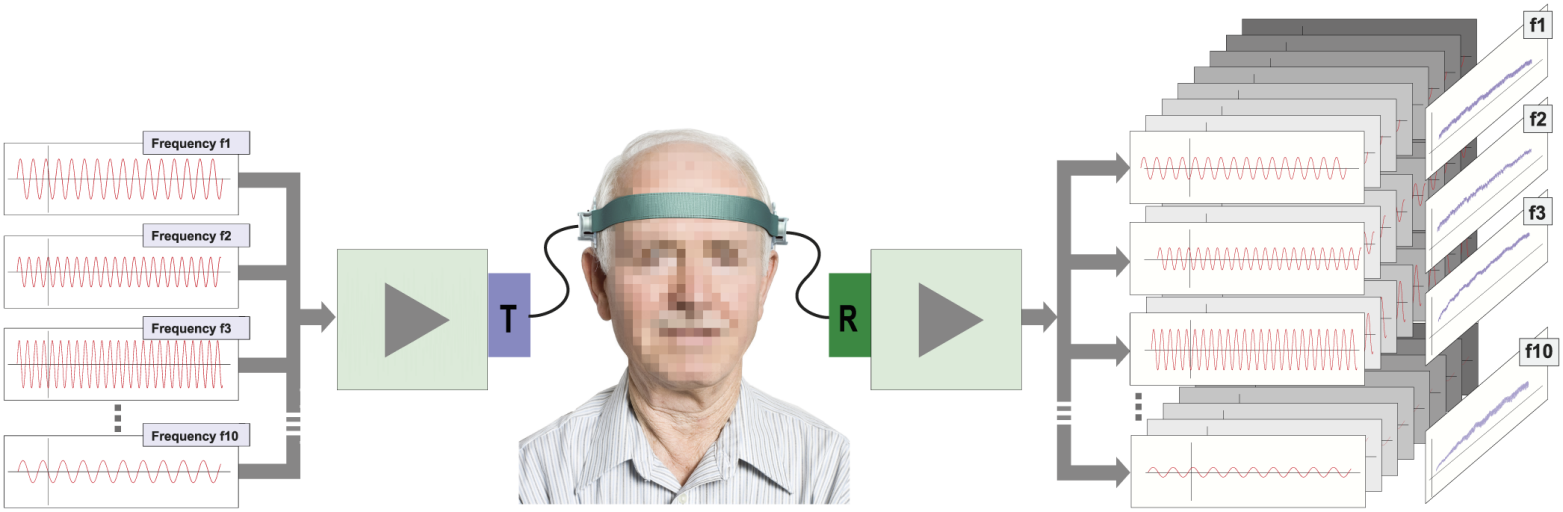
An outline of the process of forming a multidimensional phase bundle: The compound multispectral signal is being emitted at one side of a patient’s head, and after transversing skull bones and the brain tissue, it is being received on the other side. Then, the received signal is being decomposed into sine waves with base frequencies equal to the ones included in the original signal, and their phases φ1, …, φH (here H = 10) are reconstructed.

The ACG examination time is 30 seconds. We performed the examination three times for each patient, and the average value was used for further processing using the multi-spectral ultrasound brain scanner Sonovum UltraEASY^™^. (Fig 1)

### Statistical analysis

The groups of patients were compared using Fisher’s Exact Test for categorical variables, and Kruskal-Wallis-Test for quantitative variables. The statistical significance is shown using p-values. The classification was done using a gradient-boosting machine and we used 10 x 5-fold cross-validation as the validation procedure. Statistical analyses were performed using R Version 3.3.0 software with the packages gbm, plotROC and ggplot2.

## Results

Characteristics of 97 patients and cardiovascular risk profiles are shown in Table 1. Median age was 66.26 years, and 42.1% of the participants were women.

**Table 1:** Baseline characteristics.

	No Hypertension n = 22	Yes Hypertension n = 75	P–value
Age	64 (± 8)	66 (± 6)	0.26
Gender	8 (36%)	33 (44%)	0.63
Head circumference	57 (± 2)	57 (± 2)	0.38
CAD	8 (36%)	41 (55%)	0.15
Diabetes mellitus	3 (14%)	22 (29%)	0.17
Vascular diseases	1 (5%)	7 (9%)	0.68
Thyroid diseases	3 (14%)	8 (11%)	0.71
Asthma	1 (5%)	3 (4%)	1.00
Smoking	4 (18%)	8 (11%)	0.46
Fam. Stroke	5 (23%)	21 (28%)	0.79
Fam. Heart	8 (36%)	12 (16%)	0.068

CAD—coronary artery disease, Fam. Stroke—stroke in family, Fam. Heart—heart diseases in family.

**Fig 2:**
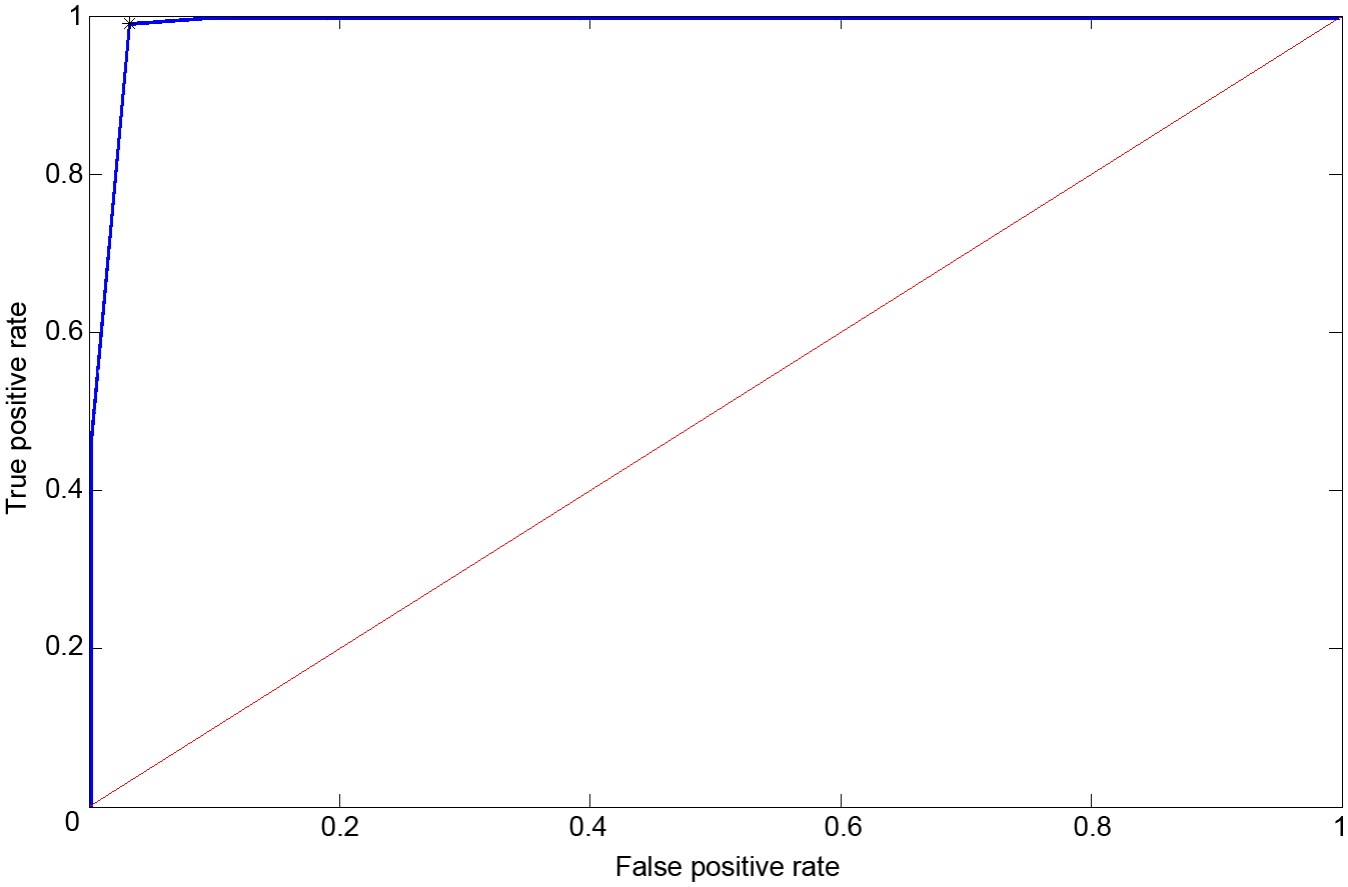
Receiver operating characteristic plot of the gbm classifications: The features of the ACG data for 97 patients with asymptomatic atrial fibrillation (40 female, 57 male, age 66.26 +- 6.54 years) were combined using a gradient boosting machine to classify if a patient has hypertension (positive result of the test) or if a patient does not have hypertension (negative result of the test). The AUC was 0.958 (sensitivity 0.99, specificity 0.968).

Besides hypertension, many of the participants, 25.8%, were diabetic and 12.4% were smokers. Overall, 26.8% of patients already had an established history of cardiovascular disease. In group I, the vast majority (82.7%) were taking ACE inhibitors, 49.3% were taking cardio-selective beta blocker, 21.3% were taking calcium channel blocker and 8.0% were on thiazide diuretics. In group II, 31.8% of patients were taking ACE inhibitors, 50.0% were taking cardio-selective beta blocker, 4.5% were taking calcium channel blocker and 4.5% were on thiazide diuretics. Vitamin K antagonist (42.7 vs 27.3), new anticoagulant (42.7 vs 27.3) and platelet aggregation inhibitors (25.3 vs 40.9) were used in group I and group II, respectively. Significant difference in treatment between these two groups was observed only for ACE inhibitors (Table 2). No significant differences in head circumference were found between the two groups. The numerical results are shown in Table 3. We have shown that ACG can be used for detection of hypertension in AF patients. After introducing a machine learning technique, the ROC plot with an AUC of 0.958 with sensitivity 0.99 and specificity 0.968 was obtained (Fig 2).

**Table 2:** Treatment data.

	No Hypertension n = 22	Yes Hypertensionn = 75	P-value
Lipid-lowering therapies	9	41	0.333
Hypoglycemic	2	9	1.000
ACE Inhibitors	7	62	>0.001
Cardioselective Beta Blocker	11	37	1.000
Statins	8	39	0.231
Anticoagulant	6	32	0.223
Platelet Aggregation Inhibitors	9	19	0.184
Vitamin K Antagonist	6	32	0.223
Group I Antiarrhythmics	8	12	0.068
Calcium Channel Blocker	1	16	0.108
Thiazide Diuretics	1	6	1.000
Non-Cardioselective Beta Blockers	1	8	0.679
Proton Pump Inhibitors	3	5	0.376

**Table 3:** Time of flight and attenuation measured for three different frequencies. The differences between the mean values of the two groups examined were checked using Kruskall-Wallis test.

	Group Hypertension		Group No Hypertension		Kruskall-Wallis Test
Variable	Mean	SD	Mean	SD	p value
ToA Freq. 2	97.71167	3.740836	98.8017	3.876631	0.04823788
ToA Freq. 10	97.51462	3.692365	98.60944	3.929389	0.05027031
ToA Freq. 6	97.65715	3.743918	98.76313	3.926999	0.05166476
ATT Freq. 10	0.4921344	0.1867929	0.5723731	0.1794155	0.002191074
ATT Freq. 9	0.5305551	0.1638107	0.5686885	0.1699262	0.076637535
ATT Freq. 4	0.1558638	0.1211638	0.1406993	0.1293839	0.249794034

## Discussion

We demonstrate that the use of multiple frequencies shows the dispersive character of brain tissue and provide new interpretations for signal changes. In non-linear material, such as biological tissue like human brain tissue, an effect of longitudinal wave dispersion can be clearly observed and measured. The non-linear, frequency-dependent bulk modulus of the medium results in different propagation speeds for different ultrasound frequencies. In addition to the observed changes in propagation speed, different attenuation profiles can also be observed. It appears that hypertension increases the stiffness of brain tissue, which is directly linked to the changing of the bulk modulus. Furthermore, some small changes in the white matter, e.g., WML, play a huge role in scattering—NOT absorbing—the ultrasound signal, which could be precisely observed in the attenuation profile for the different frequencies. In a “normal” case, the attenuation increases when ultrasound frequency increases. In our study, however, due to the very frequency-selective method provided by ACG, we are able to measure both parameters of tissues dispersivity in the form of ToA, as well as selective tissue attenuation, or scattering, in frequency-dependent ultrasound signal attenuation [19].

The first relevant finding of this study was that patients with HT had a significantly higher difference in ACG brain signals when compared to patients without HT in the AF group. The Framingham Heart Study showed that early elevation in blood pressure (prior to 50 years of age) was consistent with increasing peripheral vascular resistance [24]. As HT develops, the small brain arteries and capillaries are exposed to a high level of pressure, which results in leaking and rupturing [25]. Both cerebral microbleeds and WML are cerebral small vessel diseases. LADIS, a population-based study, suggested that HT was a risk factor for WML, but only in subjects with no history of stroke [26]. To date, MRI, CT, and positron emission tomography (PET) scans have been used to diagnose cerebral lesions in patients [27, 28]. For the first time, we have shown that ACG is an effective way to diagnose cerebral lesions and may be a tool for screening patients with HT. A group of patients with AF was selected inadvertently. AF is the most common cardiac arrhythmia, affecting an estimated 1% of the population [29]. According to the literature reports, the arrhythmia patients belong to the population group in which we can expect changes in the brain despite the absence of clinical symptoms [30, 31]. Hypertension and AF are considered the most important risk factor for developing of WML, chronic brain ischemia, silent stroke and other micro-lesion in the brain [20, 32–34].

Furthermore, CMBs occurring in the deep brain are mostly related to small vessel diseases induced by atherosclerosis or hypertension, while CMBs focused in the lobes are more likely caused by cerebral amyloid angiopathy [26]. In addition, Yamada et al. revealed a close relationship between the number of CMBs and periventricular WML, or deep WML, and regarded leukoaraiosis as an independent risk factor of CMB severity [35]. In our ACG study, based on a very accurate measurement of speed of sound and attenuation of ultrasonic complex pulses in the brain, the very small change difference in brain tissue density is sensed.

The second major finding is very high sensitivity and specificity for differentiating between AF patients with and without hypertension.

Most importantly, the selection of participants was performed randomly from a primary-care setting. The cohort was routinely treated and monitored by general practitioners, avoiding the bias that might be caused by selection in more specialized contexts, such as hospital units. The present study establishes, for the first time, that ACG can detect very small changes and differences in brain acoustical properties, when comparing non-hypertensive patients to hypertensive ones in the AF group. High sensitivity and specificity in brain signal differentiation in patients with and without hypertension is a fascinating finding that will require further testing in a larger population of patients.

The authors suppose that the major goal of ACG application was not to create a more sensitive piece of medical equipment, like MRI, but to make a semi-automatic non-invasive screening system which can save cost and time, and does not require a specially- skilled operator, such as a high-level radiologist or neurologist. This device will allow monitoring of the brain’s health by the general practitioner or cardiologist, avoiding time-consuming and expensive procedures, to check the brain’s health state on a regular basis. Acoustocerebrography is able to track the development of the brain state, almost on a daily basis.

The major limitation of our study is the lack of an MRI test showing the kinds of brain lesions that were present. However, in our previous study, we have shown a high correlation between the number of WML revealed with MRI (1.5T) and ACG signals [20]. Moreover, De Cocker et al. showed high-resolution-imaging 7T MRI is much more accurate compared to 1.5T and 3.0T imaging of the brain and its blood supply in different cerebrovascular diseases by enabling the evaluation of the brain parenchyma on a submillimeter scale, revealing very small cerebrovascular lesions, such as cortical microinfarcts [36].

Acoustocerebrography allows evaluation of the state and changes of the brain tissue and could be used for closing the existing gap in the preventive diagnosis of disorders of the central nervous system. The ACG ultrasonic method can be integrated into a mobile, easy-to-use diagnostic device that could provide a complement to the present imaging techniques. The measurement of a few acoustic parameters of cerebral tissue, such as attenuation/absorption and frequency-dependent speed of sound within the transmitted pulse frequency spectrum, prove to be specific differentiates in very high-risk patients for a cardiovascular event. Our preliminary study will be followed up for cardiac end-points, and we plan to perform a multicenter prospective study.

## Conclusion

Acoustocerebrography is a novel, promising method for discovering new markers for high-risk patients with asymptomatic AF and hypertension. The new, incremental findings presented in this study show that short and easy noninvasive examinations using ACG provides information on changes in the acoustic properties of brain tissue. It can be assumed that the marked difference in the ACG signal in patients with hypertension is due to changes in the brain tissue, and it allows for the detection of high-risk patients with asymptomatic AF and hypertension. This examination can be performed in a few minutes, potentially in any doctor’s office.
